# Supplementary material for: Amplification of electromagnetic fields by a rotating body
Source: Nat Commun. 2024 Jun 27;15:5453. doi: 10.1038/s41467-024-49689-w (PMC11211504; doi:10.1038/s41467-024-49689-w)
Supplement: Supplementary file 1 — Supplementary Information [file 41467_2024_49689_MOESM1_ESM.pdf]

SUPPLEMENTARY INFORMATION FOR AMPLIFICATION OF ELECTROMAGNETIC WAVES BY A ROTATING BODY

Abstract This supplementary information contains more details related to the main paper about the sphere model, the cylinder model, and discuss the squirrel-cage induction generator in comparison to the solid rotor case. Finally we give more details and photographs of the experimental apparatus.

THE SPHERE MODEL

We model the system composed by the LC toroid circuit and the spinning cylinder within the toroid gap as shown in Fig. 1c and 1d of the main manuscript. Amplification occurs when the resistance $\mathcal{R}$ induced by the cylinder in the circuit becomes negative. To compute the effect of the cylinder on the circuit we approximate the cylinder as a sphere with the same radius a as that of the cylinder. This is justified by the fact that the gap is quasi-cubical, so the effective length of the cylinder inside the gap is close to a. Then, we compute the magnetic dipole moment $\boldsymbol{m_0}$ induced by the magnetic field in the conductive sphere. This will allow us to calculate the magnetic flux back-reflected into the circuit, and hence the induced resistance. To do this we follow the derivation in [2], the difference lays in the magnetic field $\mathbf{B_0}$ which is oscillating rather than rotating.

Let's consider the spinning axis of the field to be oriented along z and the magnetic flux density $\boldsymbol{B_0}$ produced by the coil wrapped around the toroid to be uniform and oscillating at a frequency ω.
In the lab reference frame we can write the field in the toroid gap as:

$$\mathbf{B_0} = \beta \mathbf{b_0} I, \tag{S1}$$

where I is the current in the coil wrapped around the toroid. The geometrical factor $\beta = (0.40 \pm 0.03)$ T/A has been measured experimentally. The vector $\mathbf{b_0} = (1,0,0)^T e^{i\omega t}$ represents the linear polarization of the field oscillating at frequency ω. To our purposes it is convenient to write $\mathbf{b_0}$ as the sum of two rotating vectors, co-rotating and counter-rotating with respect to the rotor, such that:

$$\mathbf{b_0} = \frac{1}{2}\left[(1,i,0)^T + (1,-i,0)^T\right] e^{i\omega t}. \tag{S2}$$

In order to see the amplification effect we are looking for, calculations are carried out in the reference frame co-rotating with the rotor. In this frame, we write the vector $\mathbf{b_0}$ as the sum of a corotating (-) and a counter-rotating (+) terms:

$$\mathbf{b}'_{\mathbf{0}} = \frac{1}{2}\left[(1,i,0)^T e^{i\omega_- t} + (1,-i,0)^T e^{i\omega_+ t}\right] = \mathbf{b}'_- + \mathbf{b}'_+. \tag{S3}$$

so that $\mathbf{B}'_{\mathbf{0}} = \mathbf{B}'_- + \mathbf{B}'_+ = B_0\left(\mathbf{b}'_- + \mathbf{b}'_+\right)$. We define $\omega_\pm = \omega \pm \Omega$ as the corotating (ω_-) and counter-rotating (ω_+) frequencies of the field with respect to the rotor, with ω the frequency of the field $\boldsymbol{B_0}$ and Ω the rotation frequency of the rotor in the lab frame. From now on we drop the prime for simplicity, since all calculations are carried out in the rotating frame.

Our purpose is to find the form of the vector potential $\boldsymbol{\mathcal{A}}$, which allows us to compute the magnetic dipole moment induced in the rotor, and the magnetic flux $\Phi = \beta|\boldsymbol{m_0}|$ back-reflected by the magnetic dipole into the toroid circuit.

To this aim, we solve Maxwell's equations, which describe the interaction of the magnetic field $\boldsymbol{B}$ generated by the circuit and the conductive sphere and we find the equation for the vector potential [2]:

$$-\Delta\mathcal{A} + \mu_0\mu_r\sigma\frac{\partial\mathcal{A}}{\partial t} = 0, \tag{S4}$$

where σ is the electrical conductivity and μ_0 and μ_r are the vacuum and relative permeability, respectively. In order to solve this last equation for $\boldsymbol{\mathcal{A}}$, we divide the space in two regions, inside and outside the sphere with radius a, and consider the following three boundary conditions at the interface between the two regions and at infinity:

$$B_\perp^{in} = B_\perp^{out} \tag{S5}$$

$$H_\parallel^{in} = H_\parallel^{out} \tag{S6}$$

$$\mathcal{A} \underset{r\to\infty}{\longrightarrow} \mathcal{A}_\infty = \frac{1}{2}\mathbf{B_0}\times\mathbf{r} \tag{S7}$$

where $\mathbf{B} = \mu_0\mu_r\mathbf{H}$ and $\boldsymbol{r}$ is the position vector in the co-rotating reference frame centered at the center of the sphere. The magnetic field outside the sphere $\mathbf{B}^{\mathbf{out}} = \mathbf{B_0} + \mathbf{B}^{\mathbf{refl}}$ is composed of two components: the incident magnetic field generated by the circuit $\mathbf{B_0}$ plus the magnetic field reflected from the rotor $\mathbf{B}^{\mathbf{refl}}$. Let us remind that $\mathbf{B_0}$ is composed by a co-rotating and a counter-rotating component (see eq. (S3)). The magnetic field inside the rotor $\mathbf{B}^{\mathbf{in}}$ is given by the component of the incident field transmitted in the scattering.
Considering the last boundary condition, the complex vector potential $\mathcal{A}$ for a uniform applied field $\mathbf{B_0}$ can be generally written as $\mathcal{A} = \frac{1}{2}\left[F(r)\mathbf{B_0}\right] \times \mathbf{r}$ [2]. We can generalize the vector potential $\mathcal{A}$ to our case as a sum of co-rotating and counter-rotating contributions:

$$\mathcal{A} = \frac{1}{2}\left[F(\omega_-, r)\mathbf{B}_- + F(\omega_+, r)\mathbf{B}_+\right] \times \mathbf{r} = \mathcal{A}_- + \mathcal{A}_+. \tag{S8}$$

The function $F(\omega, r)$, such that $F(\omega, r \to \infty) = 1$, can be computed by substituting eq. (S8) in eq. (S4), finding [2]:

$$F\left(\omega_\pm, r\right) = 1 + D\left[c\left(\omega_\pm\right) a\right]\left(\frac{a}{r}\right)^3 \qquad \text{for} \quad r > a \tag{S9}$$

$$F\left(\omega_\pm, r\right) = \left\{1 + D\left[c\left(\omega_\pm\right) a\right]\right\}\frac{f\left[c\left(\omega_\pm\right) r\right]}{f\left[c\left(\omega_\pm\right) a\right]} \qquad \text{for} \quad r \leq a. \tag{S10}$$

where $c\left(\omega_\pm\right) = \sqrt{-i\mu_0\mu_r\sigma\omega_\pm}$.
The functions $f(x)$ and $D(x)$ are given by:

$$f(x) = \sin(x)/x^3 - \cos(x)/x^2 \tag{S11}$$

$$D(x) = \frac{(2\mu_r + 1)g(x) - 1}{(\mu_r - 1)g(x) + 1} \tag{S12}$$

(S13)

where

$$g(x) = f(x)\frac{x}{\sin(x)} = \frac{1 - x\cot(x)}{x^2}. \tag{S14}$$

These last equations define the explicit form of the vector potential $\mathcal{A} = \mathcal{A}_- + \mathcal{A}_+$ in the whole space.
It is now possible to compute the magnetic dipole moment $\mathbf{m_0}$ induced in the rotating sphere, which will be composed by two components: one co-rotating with the sphere and another one counter-rotating. From the relation between $\mathbf{m_0}$ and the reflected component of the vector potential $\mathcal{A}$

$$\mathcal{A} = \frac{\mu_0}{4\pi}\frac{\mathbf{m_0} \times \mathbf{r}}{r^3} \tag{S15}$$

we find

$$\mathbf{m_0} = \frac{\pi}{\mu_0} I a^3 \beta\left[F\left(\omega_-, a\right) - 1\right]\begin{pmatrix}1\\ i\\ 0\end{pmatrix} e^{i\omega_- t} + \frac{\pi}{\mu_0} I a^3 \beta\left[F\left(\omega_+, a\right) - 1\right]\begin{pmatrix}1\\ -i\\ 0\end{pmatrix} e^{i\omega_+} t = \tag{S16}$$

$$= \chi\left(\omega_-\right)\mathbf{B}_- + \chi\left(\omega_+\right)\mathbf{B}_+. \tag{S17}$$

It is worth noting that in order to correctly calculate $\mathbf{m_0}$, we have subtracted a factor 1 from $F(\omega_\pm, a)$ corresponding to the contribution of the incident field $\mathbf{B_0}$.
In the last equation we have defined the susceptibility :

$$\chi\left(\omega\right) = \frac{2\pi}{\mu_0} a^3\left[F\left(\omega, a\right) - 1\right], \tag{S18}$$

i.e., the complex response function of the sphere to a rotating magnetic field. Note that the susceptibility can be decomposed as $\chi = \chi' - i\chi''$, where the real part χ' represents the conservative component in-phase with the applied field while the imaginary part χ'' is out-of-phase and therefore dissipative.

Looking at the amplification from the point of view of the LC circuit, the rotating magnetic moment will couple a magnetic flux Φ into the circuit with peak value $\Phi = \beta|\mathbf{m_0}|$. We can thus write a relation $\Phi = \left[\alpha\left(\omega_-\right) + \alpha\left(\omega_+\right)\right] I$,

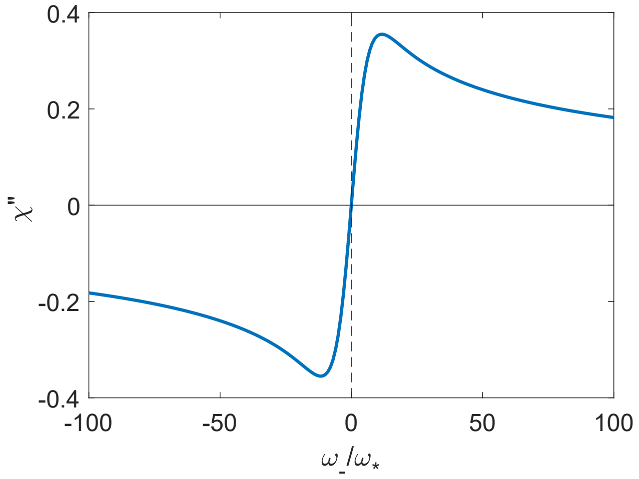

FIG. S1. Dissipative response of the sphere χ'', normalized by the factor $2\pi a^3/\mu_0$ as a function of the normalized field frequency in the corotating frame ω_-.

where the flux response function is related to the sphere susceptibility by $\alpha(\omega) = \beta^2 \chi(\omega)$. Since the flux in the laboratory frame is oscillating at ω, the associated voltage induced in the circuit is then:

$$V = i\omega\Phi = i\omega\left[\alpha(\omega_-) + \alpha(\omega_+)\right] I \tag{S19}$$

Again, we can decompose $\alpha = \alpha' - i\alpha'' = \beta^2(\chi' - i\chi'')$, so that we can define the resistance $\mathcal{R}$ and the inductance $\mathcal{L}$ generated by the presence of the rotor as:

$$\mathcal{R} = Re[V/I] = \omega\left[\alpha''(\omega_-) + \alpha''(\omega_+)\right] \tag{S20}$$

$$\mathcal{L} = Re[\Phi/I] = \left[\alpha'(\omega_-) + \alpha'(\omega_+)\right]. \tag{S21}$$

The factor α' gives the inductance $\mathcal{L}$ generated by the presence of the rotor in the gap, while $\omega\alpha''$ can be seen as a resistance $\mathcal{R}$ induced by the rotor into the circuit. Hence amplification corresponds to a negative resistance that leads to power emission into the electromagnetic (EM) mode as opposed to the expected (for a non-rotating or slowly rotating rotor) power absorbed from the EM mode. We observe that the resistance $\mathcal{R}$ is composed by a co-rotating and a counter-rotating components. We have amplification only if the co-rotating one is larger than the counter-rotating one. This is possible since in general $\alpha(\omega - \Omega) \neq \alpha(\omega + \Omega)$.

To understand this point it is instructive to look more in detail at the behaviour of the response function $\chi(\omega)$ of the cylinder with respect to the EM field. The response of the system is characterized by a length scale $\delta(\omega) = (\sigma\mu_0\mu_r\omega)^{-1/2}$, which in our model is encoded in the coefficient $c(\omega) = \sqrt{i}/\delta(\omega)$, and is usually known as the penetration depth. The magnitude of the dissipative component of the response function $|\chi''|$ (see Fig. S1) is maximized when the penetration depth approaches the system size (characterised by the sphere radius a). This means that there is a maximum absorption (positive χ'') and a maximum amplification (negative χ'') when ω_- is such that the penetration depth is of the order of the sphere radius a (we are referring to the corotating frequency ω_- being the only one to undergo negative Doppler-shift when $\Omega > \omega$, in the present experiment). Likewise, we can define a scaling frequency $\omega_* = (\mu_0\mu_r\sigma a^2)^{-1}$ at which the penetration depth equals the radius a of the sphere.

The dissipative component χ'' as a function of the normalized frequency ω_-/ω_* for a purely conductive sphere with $\mu_r = 1$ is plotted in Fig. S1. The function is odd, flipping sign when crossing 0 towards negative frequencies, which is at the core of the Zel'dovich-like amplification observed in this work. It is worth making two considerations here. First of all noticing that the dissipative response function χ'' behavior has to be considered in the corotating reference frame. Secondly we are considering it only for the frequency ω_- since it is the only one achieving negative frequencies in this system when $\Omega > \omega$. However, the total dissipative response function has two contributions, $\chi''(\omega_-) + \chi''(\omega_+)$, as shown in Eq. (S20) (and Eq. (4) in the main manuscript). Furthermore, in Fig. S1 we observe that χ'' achieves a maximum absolute value at $\omega_m = 11.6\omega_*$ ($\omega_* = 2\pi \times 8.4$ Hz in this system) and drops to 0 in both limits $|\omega| \ll \omega_m$ and $|\omega| \gg \omega_m$. For $|\omega| \ll \omega_m$ the dissipation vanishes because no eddy currents are induced in the sphere, while for $|\omega| \gg \omega_m$ eddy currents are confined to a vanishingly thin skin layer $\delta \ll a$.

When the sphere is rotating at Ω and the field is a superposition of co-rotating and counter-rotating fields, as discussed above, we have two dissipative components, shown in Figure S2, as function of the normalized rotation frequency for two representative field frequencies $\omega = 50\omega_*$ and $\omega = 500\omega_*$. The corotating term becomes negative when $\Omega > \omega$ i.e. when the corotating frequency $\omega_- = \omega - \Omega < 0$. The sum of the two terms becomes negative for Ω larger than a certain critical frequency $\Omega_c > \omega$. The relative width of of the transition becomes narrower at higher ω, however its absolute value is set by ω_m, so it is independent of ω.

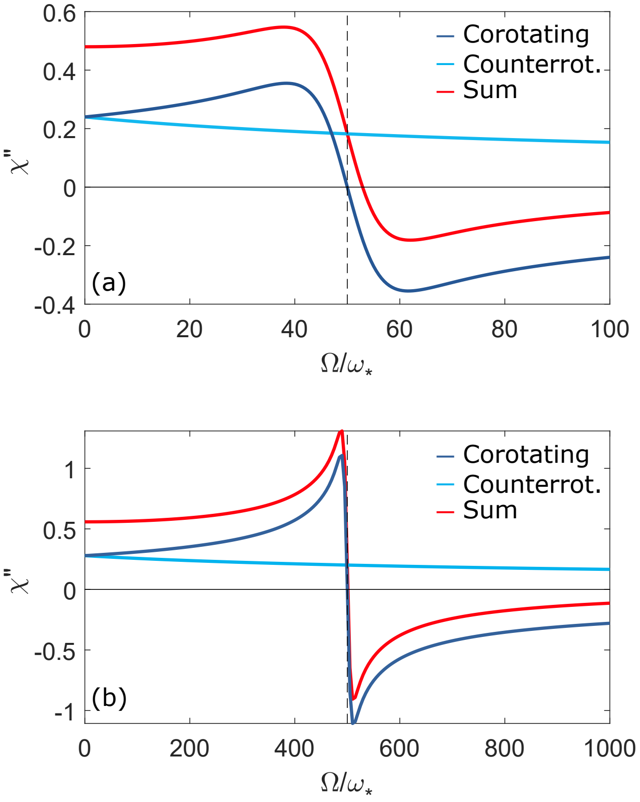

FIG. S2. (a): Dissipative response of the sphere χ'', normalized by the factor $2\pi a^3/\mu_0$, as function of normalized rotation frequency for the the two components corotating (blue line) and counterrotating (cyan line). The red line is the sum of the two terms, showing that the total dissipation can indeed become negative. Here the field frequency is set to $\omega = 50\omega_*$ (dashed vertical line). Bottom panel: the same but now for $\omega = 500\omega_*$ (dashed vertical line).

THE CYLINDER MODEL

The rotating body used for the experiment being a metallic cylinder, we also compute the amplification of a cylindrical body: we will compare this model with the sphere one and the experimental data at the end of this section. For symmetry reasons, analytical solutions in the cylindrical case exist only in the limit of infinite length. Let us consider a non rotating with infinite length in a transverse oscillating magnetic field, along x. To compute the effect of the cylinder we write the oscillating magnetic field back-reflected from the cylinder as a vector $\mathbf{B} = (B_r, B_\phi, B_z)$ in cylindrical coordinates. Considering the geometry of our system the B_z component is null since the external magnetic field is transverse to the cylinder and no induced $\hat{z}$ component is anticipated. This leaves us with a 2D problem [4]. Solving Maxwell equations for this problem gives the components of the back-reflected field from the cylinder:

$$\begin{aligned} B_r &= B_0 \tfrac{R'^2}{r^2} \cos(\phi) e^{-i\omega t} \\ B_\phi &= -B_0 \left(-\tfrac{R'^2}{r^2} \right) \sin(\phi) e^{-i\omega t}, \end{aligned} \tag{S22}$$

where (r, ϕ) are the polar coordinates in the 2D plane, B_0 is the incident field amplitude. R'^2 is given by

$$R'^2(\omega) = a^2 \frac{(\mu_r + 1)J_1(\sqrt{iR_m}) - \sqrt{iR_m}J_0(\sqrt{iR_m})}{(\mu_r - 1)J_1(\sqrt{iR_m}) - \sqrt{iR_m}J_0(\sqrt{iR_m})} \tag{S23}$$

with $R_m(\omega) = [a/\delta(\omega)]^2$ is the magnetic Reynolds number and $\delta(\omega) = 1/\sqrt{\mu_0\mu_r\sigma\omega}$ is the penetration depth, depending on the frequency of oscillation of the magnetic field ω [4]. σ is the cylinder conductivity and μ_0 and μ_r are the vacuum and relative permeabilities. The functions J_0 and J_1 are Bessel functions of the first kind. Let us consider the case of rotating cylinder. Equations (S22) needs to account the rotation of the cylinder at frequency Ω. In the reference frame co-rotating with the cylinder, the co-rotating and a counter-rotating components of the oscillating magnetic field rotate at the frequencies $\omega_- = \omega - \Omega$ and $\omega_+ = \omega + \Omega$ respectively. To include the rotation we observe that the field in eq. (S22) corresponds to a linearly polarized field expressed in polar coordinates [1, 3] In polar coordinates, right and left rotating vectors can be written as

$$|\text{right}\rangle = \frac{1}{\sqrt{2}}\begin{pmatrix} \cos(\phi) - i\sin(\phi) \\ -\sin(\phi) - i\cos(\phi) \end{pmatrix} \quad \text{and} \quad |\text{left}\rangle = \frac{1}{\sqrt{2}}\begin{pmatrix} \cos(\phi) + i\sin(\phi) \\ -\sin(\phi) + i\cos(\phi) \end{pmatrix}. \tag{S24}$$

We can hence express the field in (S22) as a sum of co-rotating (right) and counter-rotating (left) components. Importantly, in this frame the frequency of the co-rotating field is different from the frequency of the counter-rotating component, being $\omega_- = \omega - \Omega$ and $\omega_+ = \omega + \Omega$ respectively. The back-reflected field for our system is

$$B_r = B_0\Big[\frac{R'^2(\omega_-)}{2r^2}\Big(\cos(\phi) - i\sin(\phi)\Big)e^{-i\omega_- t} + \frac{R'^2(\omega_+)}{2r^2}\Big(\cos(\phi) + i\sin(\phi)\Big)e^{-i\omega_+ t}\Big] \tag{S25}$$

$$B_\phi = -B_0\Big[\frac{R'^2(\omega_-)}{2r^2}\Big(-\sin(\phi) - i\cos(\phi)\Big)e^{-i\omega_- t} + \frac{R'^2(\omega_+)}{2r^2}\Big(-\sin(\phi) + i\cos(\phi)\Big)e^{-i\omega_+ t}\Big]. \tag{S26}$$

Similarly to the sphere model we now compute the equivalent magnetic moment induced in the cylinder. To this end, we write the $\boldsymbol{B}$ field generated by a linear distribution of magnetic dipole moment with density per unit length $\boldsymbol{\lambda}$ along the cylinder axis, that is:

$$\mathbf{B}(r, \phi) = \int_{-\infty}^{\infty} \frac{\mu_0}{4\pi}\Big(\frac{3\mathbf{r}(\boldsymbol{\lambda}\cdot\mathbf{r})}{r^5} - \frac{\boldsymbol{\lambda}}{r^3}\Big)dz, \tag{S27}$$

where $\mathbf{r}$ is the position vector in cylindrical coordinates. The solution of this equation is:

$$\mathbf{B}(r, \phi) = \Big(\frac{\mu_0}{2\pi}\frac{\lambda_r}{r^2}, -\frac{\mu_0}{2\pi}\frac{\lambda_\phi}{r^2}\Big)^T. \tag{S28}$$

Comparing eqs. (S28) and (S25) we find the expression for the magnetic moment density $\boldsymbol{\lambda} = (\lambda_r, \lambda_\phi)^T$ being

$$\lambda_r = \frac{2\pi r^2}{\mu_0}\frac{1}{2r^2}B_0\Big[R'^2(\omega_-)\Big(\cos(\phi) - i\sin(\phi)\Big)e^{-i\omega_- t} + R'^2(\omega_+)\Big(\cos(\phi) + i\sin(\phi)\Big)e^{-i\omega_+ t}\Big] = \tag{S29}$$

$$\lambda_\phi = \frac{2\pi}{\mu_0}B_0\frac{1}{2}\Big[R'^2(\omega_-)\Big(-\sin(\phi) - i\cos(\phi)\Big)e^{-i\omega_- t} + R'^2(\omega_+)\Big(-\sin(\phi) + i\cos(\phi)\Big)e^{-i\omega_+ t}\Big]. \tag{S30}$$

Now, for the actual finite case we take the solution for the density of magnetic moment of the infinite case and integrate over the finite length of the gap L, where the $\boldsymbol{B}$ field is non negligible. This gives the total effective magnetic moment induced in the finite cylinder, neglecting border effects:

Being $\mathbf{m} = \chi(\omega_-)\mathbf{B}_- + \chi(\omega_+)\mathbf{B}_+$, we find:

$$\chi(\omega_-) = \frac{2\pi}{\mu_0}R'^2(\omega_-)L \tag{S31}$$

$$\chi(\omega_+) = \frac{2\pi}{\mu_0}R'^2(\omega_+)L. \tag{S32}$$

The resistance $\mathcal{R}$ and inductance $\mathcal{L}$ generated by the presence of the rotating cylinder can be written as

$$\mathcal{R} = \omega\beta^2[\chi''(\omega_-) + \chi''(\omega_+)] \tag{S33}$$

$$\mathcal{L} = \beta^2[\chi'(\omega_-) + \chi'(\omega_+)]. \tag{S34}$$

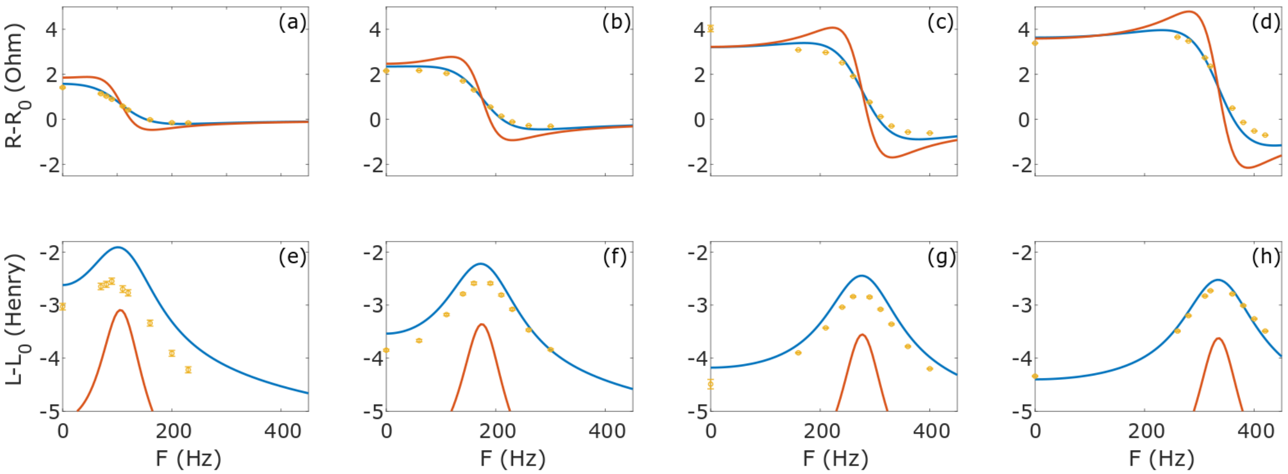

FIG. S3. Comparison of the resistance and inductance calculated through the sphere (blue) and cylinder (orange) models. $\mathcal{R} = R - R_0$ (a,b,c,d) and $\mathcal{L} = L - L_0$ (e,f,g,h) computed as function of the rotational frequency $F = \Omega/(2\pi)$ for different resonance frequencies $f = \omega/(2\pi) = [107(\text{a,e}), 175(\text{b,f}), 277(\text{c,g}), 335(\text{d,h})]$ Hz of the LC oscillator.

Figure S3 shows a comparison between the experimental results shown in Fig. 3 of the main paper, with both the sphere and the infinite cylinder model. Clearly, the sphere model captures the experimental behaviour more accurately. We attribute this experimental finding to the fact that the assumption of uniform applied field is more explicitly broken in the case of the infinite cylinder, due to the fall-off of the field at the edge of the gap. This leads to an overestimation of the rotor-induced effect. Conversely, in the sphere approximation the contributions at the edge of the gap are intrinsically suppressed by the spherical geometry.

SQUIRREL-CAGE INDUCTION GENERATOR VS SOLID ROTOR

Induction machines are a type of rotating machine where a stator circuit - the non-rotating part, usually embedded in a hollow cylinder - has alternating currents oscillating at a single frequency ω_{ac} - the frequency of the power supply, which are used to generate a rotating field. The stator can have multiple magnetic poles p, hence the frequency of rotation of the stator magnetic field is $\omega_s = \omega_{ac}/p$. Secondary circuits are mounted on the rotor, placed inside the

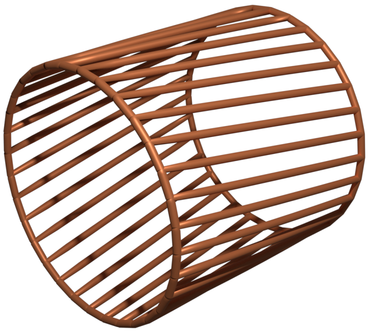

FIG. S4. Sketch of copper squirrel cage rotor.

hollow stator cylinder, and rotate at frequency ω_m - the mechanical rotation. The mechanism is based on the principle of induction: the rotor circuits are short-circuited and their currents are induced by the stator rotating field.

In most induction machines, a squirrel cage rotor is used. This is made of a solid cylinder of iron or steel carved by conductor bars, usually made of copper or aluminium, that are short-circuited by a conductive ring around the

cylinder bases (see an sketch of the conductive bars in the typical squirrel cage circuit in Figure S4). The thickness and the shape of these conductive bars determines the inductive and resistive properties of the rotor circuits. In our analysis we consider the induction system described in [5] and we write the voltage V_s induced by the stator on the rotor as

$$V_s = \frac{\omega_s^2 M^2 (R_{rot}/s)}{(R_{rot}/s)^2 + \omega_s^2 L_{rot}^2} I_s + i \frac{\omega_s^3 M^2 L_{rot}}{(R_{rot}/s)^2 + \omega_s^2 L_{rot}^2} I_s, \tag{S35}$$

where $s = (\omega_s - \omega_m)/\omega_s$ is the so called slip, that can be related to the Zel'dovich condition considering the correspondence $p \to \ell$ ($\ell = 1$ in our case), $\omega_m \to \Omega$ and $\omega_{ac} \to \omega$. M is the mutual inductance, while R_{rot}, L_{rot} are the resistance and inductance of the rotor's circuits.
It is possible to find the effective susceptibility χ_{eff} of the squirrel cage induction generator by recalling that $\alpha = \beta^2 \chi_{\text{eff}}$. The geometrical factor β for the squirrel cage circuit is just the mutual inductance M. After some manipulation, we find:

$$\chi_{\text{eff}} = \frac{\alpha}{M^2} = \frac{L_{rot}(\omega - \Omega)^2}{R_{rot}^2 + L_{rot}^2(\omega - \Omega)^2} - i \frac{R_{rot}(\omega - \Omega)}{R_{rot}^2 + L_{rot}^2(\omega - \Omega)^2}, \tag{S36}$$

where the first term on the right hand side is the inductive component (χ'_{eff}) and the second is the resistive component (χ''_{eff}), which indeed flips sign if the slip frequency $(\omega - \Omega) < 0$, i.e. the Zel'dovich condition is fulfilled.

EXPERIMENTAL APPARATUS

Fig. S5 shows photos of the experimental apparatus used to perform the experiment. The magnetic field is generated by a coil wound around a gapped toroidal ferrite core with square section 4×4 cm. An aluminum cylinder with radius $a = 2$ cm, mounted on a brush-less motor is fixed into the 4.4 cm gap in the ferrite core. As the cylinder can spin up to several hundred Hertz about its symmetry axis, protective clear plastic is mounted around it. The coil has 2×10^4 turns of 0.2 mm diameter copper wire, and is connected in series, which create a resonant circuit with the coils and improve the accuracy of measurements.

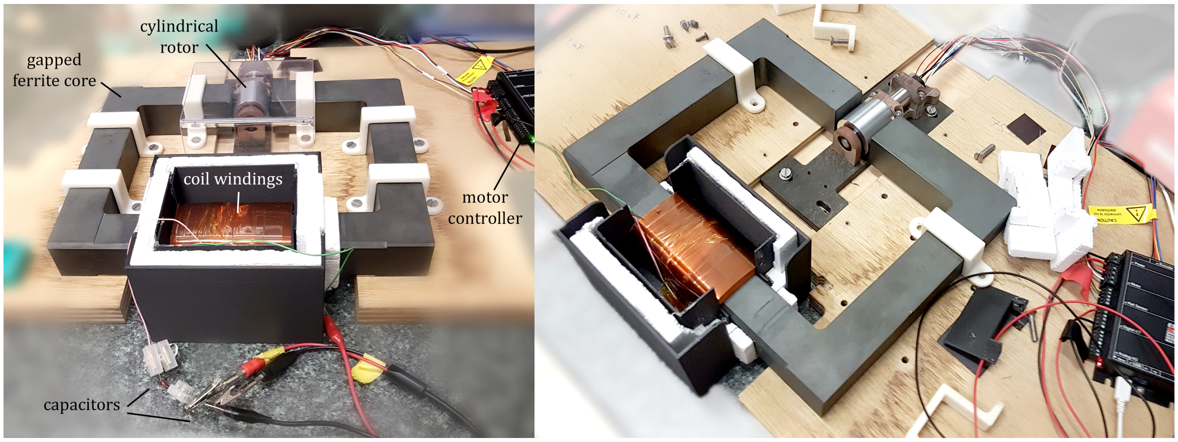

FIG. S5. The experimental apparatus used to perform the experiment. The cylindrical rotor is mounted into the gap in the toroid core, and can be driven to spin around its symmetry axis by a motor and controller. The cylinder can be removed for calibration measurements. Capacitors are connected in series with the coil windings.

[1] The linearly polarized vector in (x, y) coordinates

$$\begin{pmatrix} 1 \\ 0 \end{pmatrix} \tag{S37}$$

corresponds to the vector

$$\begin{pmatrix} \cos(\phi) \\ -\sin(\phi) \end{pmatrix} \tag{S38}$$

in polar coordinates[3].
[2] M.C. Braidotti, A. Vinante, G. Gasbarri, D. Faccio, and H. Ulbricht. Zel'dovich amplification in a superconducting circuit. *Phys. Rev. Lett.*, 125:140801, 2020.
[3] Shigeki Matsuo. Matrix calculus for axially symmetric polarized beam. *Opt. Express*, 19(13):12815–12824, 2011.
[4] Michel P. Perry and Thomas B. Jones. Eddy current induction in a solid conducting cylinder with a trasverse magentic field. *IEEE Transactions on Magnetics*, 14(4):227, 1978.
[5] H. H. Woodson and J.R. Melcher. *Electromechanical Dynamics.* John Wiley and Sons, New York, NY, 1968.
